# 紫草油有效成分的高效液相色谱测定法及其在超临界流体萃取制备紫草油中的应用

**DOI:** 10.3724/SP.J.1123.2020.12009

**Published:** 2021-07-08

**Authors:** Jie SHEN, Wei SHEN, Xue CAI, Jingxia WANG, Minxia ZHENG

**Affiliations:** 1.浙江中医药大学附属第一医院, 浙江 杭州 310006; 1. The First Affiliated Hospital of Zhejiang Chinese Medical University, Hangzhou 310006, China; 2.浙江工业大学分析测试中心, 浙江 杭州 310014; 2. Analysis and Test Center, Zhejiang University of Technology, Hangzhou 310014, China

**Keywords:** 高效液相色谱, 超临界流体萃取, 二极管阵列检测, 活性成分, 紫草油, 紫草, 工艺优化, high performance liquid chromatography (HPLC), supercritical fluid extraction (SFE), photodiode array detection (PAD), active components, lithospermum oil, Lithospermum erythrorhizon, process optimization

## Abstract

紫草提取制备成的紫草油能够预防及治疗婴儿尿布疹、皮肤溃烂、湿疹等多种皮肤疾患,临床应用非常广泛,超临界流体萃取是紫草有效成分提取的优选方法。该文建立了紫草油有效成分的高效液相色谱(HPLC)测定方法,并以紫草油所含的有效成分含量为评价指标,采用三因素三水平正交试验法对紫草超临界流体萃取制备过程中的几个重要因素(萃取压力、萃取温度和CO_2_流量)进行考察,确定了紫草超临界流体萃取的最佳工艺流程。所建立的HPLC方法如下:Diamonsil C_18_色谱柱(250 mm×4.6 mm, 5 μm),流动相为含0. 1%甲酸的乙腈-含5 mmol/L甲酸铵的0.1%甲酸水溶液(75:25, v/v),等度洗脱,流速为1 mL/min;进样量为15 μL;柱温为室温;二极管阵列检测器(PAD)检测波长为275 nm。该法可在30 min内同时测定紫草油中的有效成分紫草素、乙酰紫草素、*β*-乙酰氧基异戊酰阿卡宁、异丁酰紫草素、*β*,*β*-二甲基丙烯酰紫草素和2-甲基丁酰基紫草素的含量,方法精密度、准确度、重复性好。在萃取压力23 MPa、萃取温度40 ℃、CO_2_流量27 L/h这一优化工艺流程下得到的紫草油有效成分含量最高。所建立的HPLC-PAD法简便可行、准确可靠,可用于超临界流体萃取制备紫草油的工艺过程优化和质量控制,也可为紫草及其制剂的质量评价提供参考。优化后的工艺条件能够满足紫草油制备需求,也符合生产实际需求。

## Abstract (en)

Lithospermum erythrorhizon has the functions of cooling blood, activating blood, as well as detoxifying and penetrating rash. Lithospermum oil extracted from Lithospermum erythrorhizon can prevent and treat diaper rash, skin ulceration, eczema, and other skin diseases. Supercritical fluid extraction is the optimal method for the extraction of active components from lithospermum. In this study, an analytical method was established for simultaneously determination of six active components in lithospermum oil with high performance liquid chromatography (HPLC), and the contents of the active components as the evaluation index were used to investigate several important factors in the preparation of lithospermum oil by supercritical fluid extraction. The optimized HPLC conditions were as follows: separation column, Diamonsil C_18_ (250 mm×4.6 mm, 5 μm); mobile phases, acetonitrile containing 0.1% (v/v) formic acid-0.1% (v/v) formic acid aqueous solution containing 5 mmol/L ammonium formate (75:25, v/v); flow rate, 1 mL/min; injection volume, 15 μL; room temperature; photodiode array detector (PAD); detection wavelength, 275 nm. The supercritical fluid extraction was optimized for ensuring stability of the amounts of effective components and the reliability of the quality of lithospermum oil. This will serve as the basis for preparation and quality control processes. Three factors and three levels orthogonal tests were adopted to investigate the important factors, viz. the pressure, temperature and CO_2_ flow rate in the preparation of lithospermum oil. The results showed that the developed HPLC-PAD method can be used for the simultaneous determination of shikonin, acetylshikonin, *β*-acetoxyisovaleryl akanin, isobutyryl shikonin, *β*,*β*-dimethylacryl shikonin, and 2-methylbutyryl shikonin in 30 min. The method has good precision, accuracy and repeatability. The contents of the active components were the highest when the extraction pressure, extraction temperature, and CO_2_ flow rate were 23 MPa, 40 ℃, and 27 L/h, respectively. The optimized conditions are suitable for the preparation and actual production of lithospermum oil. The HPLC-PAD method is simple, feasible, accurate, and reliable. It can be used for the preparation and quality control of lithospermum oil by supercritical fluid extraction. Thus, with this method, the stability of the contents of active ingredients and the reliability of the quality of lithospermum oil can be ensured; moreover, safe and effective drug use can be realized. The established method has obvious advantages over the traditional process and is a good candidate for widespread use.

紫草(Lithospermum erythrorhizon)是皮肤科常用的中药之一,具有凉血、活血、解毒透疹的功效。《本草纲目》记载:“紫草治斑疹、痘毒,活血凉血,利大肠”,用于血热毒盛、斑疹紫黑、麻疹不透、湿疹、水火烫伤等。由紫草提取制备成的紫草油能够预防及治疗婴儿尿布疹、皮肤溃烂、湿疹等多种皮肤疾患,临床应用非常广泛^[1]^。

紫草主要的活性成分是萘醌类化合物,包括紫草素(shikonin)、乙酰紫草素(acetylshikonin)、*β*,*β*-二甲基丙烯酰紫草素(*β*,*β*-dimethylacrylshikonin)等成分^[2]^,传统的提取紫草有效成分的方法主要包括有机溶剂萃取法、水蒸气蒸馏法、减压蒸馏法、油浸法等,其工艺复杂,产品纯度不高,常用的有机溶剂提取法提取物中常含有微量有机溶剂残留。超临界流体萃取(supercritical fluid extraction, SFE)是近几十年发展起来的一种新型提取技术,对许多物质有较好的渗透性和较强的溶解能力,且萃取物无溶剂残留,保证产品100%的纯天然性。梁瑞红等^[3]^对新疆紫草进行了超临界流体萃取工艺研究并与有机溶剂萃取的结果进行了比较,超临界流体萃取比有机溶剂萃取能得到更多紫草萘醌类组分,且超临界流体萃取所含的杂质更少,超临界流体萃取可作为紫草有效成分提取的优选方法。目前国内外文献报道超临界提取紫草的研究所涉及的内容主要有紫草有效成分的分离、纯化研究^[4]^、CO_2_超临界流体萃取紫草有效成分的稳定性研究^[5]^、超临界流体萃取紫草的有效成分的抗氧化研究^[6]^等。尚未见高效液相色谱同时测定紫草油中多种有效成分含量及用于超临界流体萃取制备紫草油工艺优化的研究报道,还没有形成一套严谨规范的工艺流程。调研国内外关于紫草有效成分检测的文献报道,大多是对左旋紫草素^[7,8,9,10]^、*β*,*β*-二甲基丙烯酰阿卡宁^[7,8,9]^、乙酰紫草素^[9,10]^、*β*-乙酰氧基异戊酰紫草素^[10]^、*β*,*β*-二甲基丙烯酰紫草素^[10]^、蒽醌类^[11]^2到3种有效成分的含量检测或指纹图谱研究。

本研究建立了紫草油6种有效成分含量检测的分析方法,并以紫草油所含的有效成分含量为评价指标,优化紫草超临界流体萃取制备过程中的萃取压力、萃取温度和CO_2_流量3个制备工艺条件,确定了紫草超临界流体萃取的最佳工艺流程,从而保证有效成分含量的稳定性和紫草油质量的可靠性,达到安全、有效用药的目的。

## 1 实验部分

### 1.1 仪器、试剂和材料

Waters 2695高效液相色谱系统(Waters, USA),配四元梯度泵、在线脱气机、Waters 2996二极管阵列检测器(PDA)、Chem Station工作站控制系统;KQ-100DA型数控超声波清洗器(昆山舒美超声仪器有限公司); EL204电子分析天平(梅特勒-托利多上海有限公司); MinispanPlus台式高速离心机(杭州奥盛仪器有限公司); Milli-Q超纯水系统(Millipore, USA)等。

紫草素(纯度>99.0%)、乙酰紫草素(纯度>99.0%)、*β*,*β*-二甲基丙烯酰紫草素(纯度>99.0%)标准品购自国家标准物质中心,*β*-乙酰氧基异戊酰阿卡宁(纯度>98.0%)、异丁酰紫草素(纯度>98.0%)标准品购自上海源叶生物科技有限公司,2-甲基丁酰基紫草素(纯度>98.0%)标准品购于上海阿拉丁生化科技有限公司;甲酸(色谱纯,上海阿拉丁生化科技股份有限公司);乙腈(色谱纯,德国默克公司);甲酸铵(分析纯,国药集团化学试剂有限公司);Milli-Q超纯水。

新疆紫草*Arnebia euchroma* (Royle) Johnst由浙江中医药大学中药饮片厂提供,由浙江中医药大学附属第一医院中药房郑敏霞主任中药师鉴定。

### 1.2 色谱条件

Diamonsil C_18_色谱柱(250 mm×4.6 mm, 5 μm),流动相:含0.1%甲酸的乙腈-含5 mmol/L甲酸铵的0.1%甲酸水溶液(75:25, v/v),等度洗脱,流速为1 mL/min;进样量为15 μL;柱温为室温;检测波长为275 nm。

### 1.3 标准溶液制备

精密称取6种紫草素标准品适量,用乙腈-水溶液(75:25, v/v)溶解,并定容于10 mL容量瓶中,配制成混合标准品储备液,于4 ℃避光保存。储备液中紫草素、乙酰紫草素、*β*-乙酰氧基异戊酰阿卡宁、异丁酰紫草素、*β*,*β*-二甲基丙烯酰紫草素、2-甲基丁酰基紫草素的质量浓度分别为100、500、400、600、500、1000 μg/L。

分别精密吸取储备液适量,并稀释成系列浓度的标准溶液,各标准系列质量浓度如下:紫草素10、20、30、40、50 μg/L,乙酰紫草素50、100、150、200、250 μg/L, *β*-乙酰氧基异戊酰阿卡宁40、80、120、160、200 μg/L,异丁酰紫草素60、120、180、240、300 μg/L, *β*,*β*-二甲基丙烯酰紫草素0、100、150、200、250 μg/L, 2-甲基丁酰基紫草素100、200、300、400、500 μg/L。

### 1.4 供试品溶液制备

取紫草超临界流体萃取所得的样品1 mg,精密称定,溶于1 mL乙腈-水(75:25, v/v)溶液中,超声1 min,用0.45 μm滤膜过滤,滤液作为供试品溶液,供分析用。

### 1.5 超临界流体萃取制备紫草油

选取新疆紫草,碾碎,粗粉碎物料过筛。紫草装料量250 g, CO_2_超临界流体萃取时间2 h,无夹带剂,一级分离,萃取压力23 MPa,萃取温度40 ℃, CO_2_流量27 L/h。

## 2 结果和讨论

### 2.1 HPLC方法的建立

2.1.1 有效成分检测波长的选择

《中国药典》记载紫草有效成分的检测波长为516 nm和275 nm^[7]^,部分文献报道紫草有效成分的检测波长为516 nm^[11]^,我们对紫草油的6种有效成分进行了光谱扫描,结果如图1所示,可以看出在275 nm和516 nm处,紫草油的6种有效成分均有较高的吸收度,但在275 nm处,6种有效成分的吸收度更高,因此选择275 nm作为检测波长。

**图 1:**
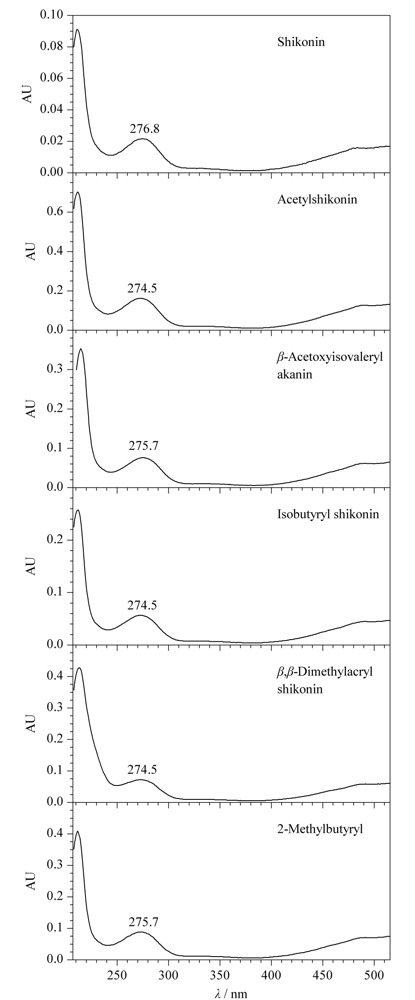
紫草油中6种有效成分的PAD光谱图

2.1.2 HPLC流动相的优化

色谱条件优化过程中,对流动相溶剂(甲醇、乙腈)、流动相酸度(0.25%、0.05%、0.1%、0.2%)和流速(0.8、1.0、1.2 mL/min)进行了考察。结果发现,乙腈对紫草油有效成分的洗脱能力强于甲醇,使得分离时间明显缩短,而且分离效果也比较好;流动相的酸度对紫草油有效成分的分离度有一定的影响,随着酸度的增大,色谱峰的峰形得到改善,但是考虑到过高的酸度可能对色谱柱的使用寿命产生影响,所以最终选择酸度为0.1%;流动相流速选择1.0 mL/min最为适合。色谱条件优化后的紫草油标准品和样品色谱图见图2。

**图 2:**
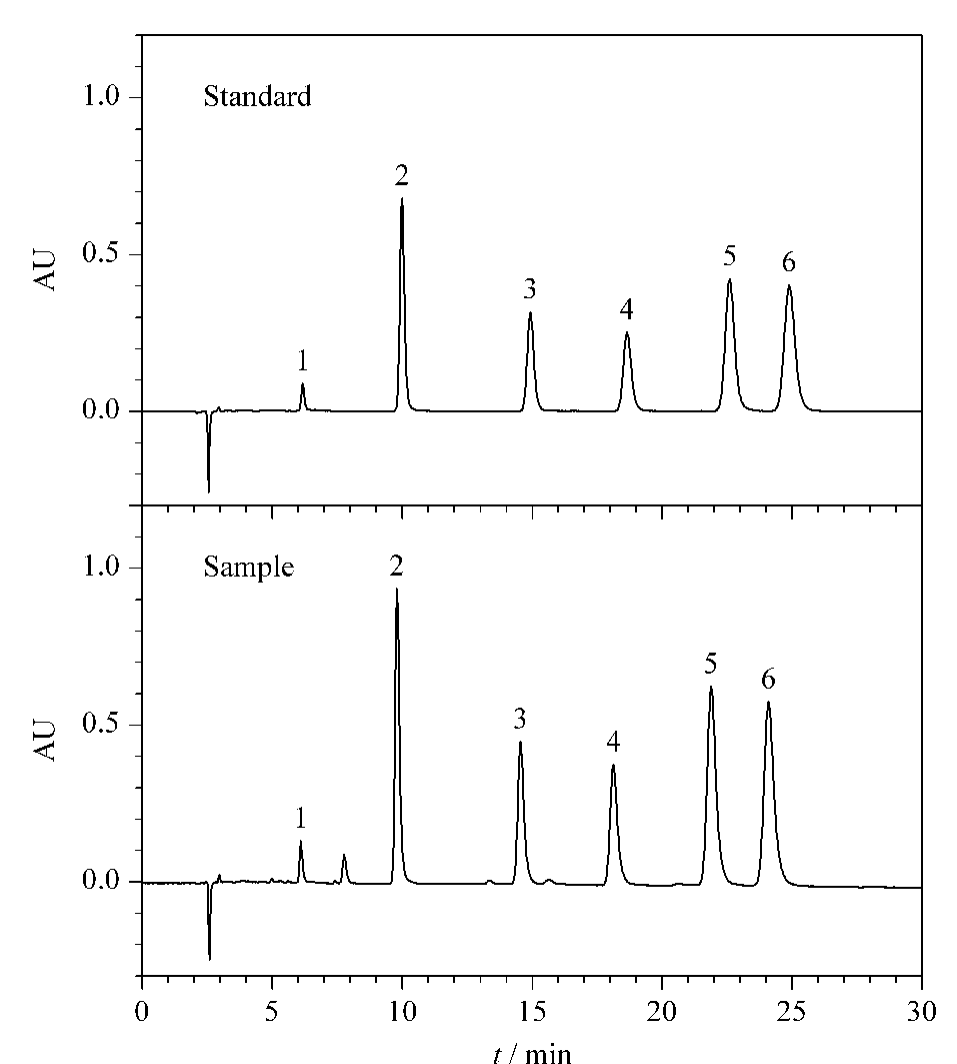
优化条件下紫草油标准品和样品的HPLC谱图

### 2.2 HPLC方法学考察

采用优化后的液相色谱条件,进样分析一系列浓度的混合标准溶液,以峰面积为纵坐标,质量浓度为横坐标,绘制标准曲线,得到线性回归方程,如表1所示,线性关系良好。信噪比(*S/N*)=3的进样质量浓度即检出限(LOD), *S/N*=10的进样质量浓度即定量限(LOQ),结果如表1所示,方法灵敏度高。

**表 1:** 6种有效成分的线性关系、检出限和定量限(*n*=3)

Compound	t_R_/min	Linear range/(μg/L)	Calibration curve	r	LOD/(μg/L)	LOQ/(μg/L)
Shikonin	6.08±0.01	10.00-50.00	Y=79583X-90506	0.9990	1.73	5.78
Acetylshikonin	9.76±0.02	50.00-250.00	Y=84361X+139619	0.9997	0.75	4.08
β-Acetoxyisovaleryl akanin	14.45±0.01	40.00-200.00	Y=71421X-81331	0.9999	2.24	6.38
Isobutyryl shikonin	18.03±0.03	60.00-300.00	Y=81910X-7028.4	0.9985	1.58	6.55
β,β-Dimethylacryl shikonin	21.81±0.05	50.00-250.00	Y=109607X-699084	0.9990	2.63	7.67
2-Methylbutyryl	24.28±0.05	100.00-500.00	Y=75658X-935990	0.9991	4.62	20.5

* *Y*: peak area of each component; *X*: mass concentration, μg/L.

配制1份混合标准溶液,混合标准溶液中紫草素、乙酰紫草素、*β*-乙酰氧基异戊酰阿卡宁、异丁酰紫草素、*β*,*β*-二甲基丙烯酰紫草素、2-甲基丁酰基紫草素质量浓度分别为30、150、120、180、150、300 μg/L,同一天内取样6次进样分析,计算日内精密度;配制1份混合标准溶液,混合标准品中各成分紫草素、乙酰紫草素、*β*-乙酰氧基异戊酰阿卡宁、异丁酰紫草素、*β*,*β*-二甲基丙烯酰紫草素、2-甲基丁酰基紫草素质量浓度分别为40、200、160、240、200、400 μg/L,连续6日每日进样分析,计算日间精密度,结果如表2所示,精密度良好。

**表 2:** 日内和日间精密度试验结果(*n*=6)

Compound	Intra-day precision			Inter-day precision	
	Content/ (μg/L)	RSD/ %		Content/ (μg/L)	RSD/ %
Shikonin	29.35	2.17		38.89	3.70
Acetylshikonin	150.29	0.19		197.06	1.47
β-Acetoxyisovaleryl akanin	120.41	0.35		160.54	3.41
Isobutyryl shikonin	186.86	3.81		231.60	4.49
β,β-Dimethylacryl shikonin	148.28	1.14		193.46	3.27
2-Methylbutyryl	293.96	2.01		393.40	2.65

按供试品溶液制备方法制备6份供试品溶液进样分析评估重复性,结果见表3。按供试品溶液制备方法制备1份供试品溶液,分别于0、2、4、8、16、24 h进样分析评估稳定性,结果见表3。

**表 3:** 重复性和稳定性试验结果(*n*=6)

Compound	Repeatability RSD/%	Stability RSD/%
Shikonin	1.92	0.36
Acetylshikonin	0.33	1.50
β-Acetoxyisovaleryl akanin	0.80	1.57
Isobutyryl shikonin	0.55	2.16
β,β-Dimethylacryl shikonin	0.52	2.49
2-Methylbutyryl	0.33	3.26

按加样回收率测定方法,分别在已知含量的样品中,加入一定质量浓度的混合标准溶液,按照供试品溶液制备方法制备供试品6份,进样分析,计算加样回收率,结果见表4。

**表 4:** HPLC分析方法的加样回收率结果(*n*=6)

Compound	Background/ (μg/L)	Added/ (μg/L)	Found/ (μg/L)	Recovery/ %	RSD/ %
Shikonin	10.27	8.50	18.15	92.68	4.25
	10.27	10.00	20.13	98.58	
	10.27	12.50	22.85	100.65	
Acetylshikonin	86.63	70.00	152.95	94.74	5.35
	86.63	86.50	171.31	97.89	
	86.63	104.00	195.93	105.09	
β-Acetoxyisovaleryl	71.42	57.50	123.43	90.45	4.19
akanin	71.42	71.50	141.70	98.29	
	71.42	85.50	153.08	95.50	
Isobutyryl shikonin	63.14	50.50	111.68	96.12	7.16
	63.14	63.00	132.08	109.42	
	63.14	75.50	145.26	108.77	
β,β-Dimethylacryl	75.81	60.50	132.57	93.81	8.35
shikonin	75.81	75.50	149.23	97.24	
	75.81	90.50	175.10	109.71	
2-Methylbutyryl	148.17	118.50	251.29	87.02	4.76
	148.17	148.00	289.20	95.29	
	148.17	178.00	314.86	93.64	

以上结果证明该分析方法的重复性、稳定性和准确度良好。此分析方法可用于紫草油中6种有效成分的定量分析,有效成分的含量指标可应用于评价超临界流体萃取制备紫草油工艺的优化过程。

### 2.3 超临界流体萃取制备紫草油的工艺条件筛选

选取新疆紫草,碾碎,粗粉碎物料过筛。每组实验装料量250 g, CO_2_超临界流体萃取时间2 h,无夹带剂,一级分离,采用三因素三水平正交试验法,正交试验因素水平见表5,实验组别见表6,考察萃取压力、萃取温度和CO_2_流量对紫草有效成分含量的影响。9组试验所得的紫草油样品通过上述已建立的HPLC分析方法检测有效成分含量,有效成分含量结果见表7,HPLC谱图见图3。

**表 5:** 正交试验因素水平表

Level	Extraction pressure/MPa	Extraction temperature/℃	CO_2_ flow/ (L/h)	Blank control
1	18	30	27	1
2	23	35	30	2
3	28	40	32	3

**表 6:** 超临界流体萃取制备紫草油工艺优化的正交试验表

Test No.	Extraction pressure	Extraction temperature	CO_2_ Flow	Blank control
1	1(18 MPa)	1(30 ℃)	1(27 L/h)	1
2	1(18 MPa)	2(35 ℃)	2(30 L/h)	2
3	1(18 MPa)	3(40 ℃)	3(32 L/h)	3
4	2(23 MPa)	1(30 ℃)	2(30 L/h)	3
5	2(23 MPa)	2(35 ℃)	3(32 L/h)	1
6	2(23 MPa)	3(40 ℃)	1(27 L/h)	2
7	3(28 MPa)	1(30 ℃)	3(32 L/h)	2
8	3(28 MPa)	2(35 ℃)	1(27 L/h)	3
9	3(28 MPa)	3(40 ℃)	2(30 L/h)	1

**表 7:** 9组超临界流体萃取工艺制备所得紫草油的有效成分含量(*n*=3)

Test No.	Shikonin	Acetylshikonin	β-Acetoxyisovaleryl akanin
1	10.90±0.29	93.85±6.54	88.18±5.94
2	11.71±0.12	109.76±4.09	93.64±2.18
3	13.98±0.27	155.94±8.15	129.43±3.77
4	13.79±0.26	137.60±0.46	113.74±0.91
5	20.54±0.39	172.14±3.02	144.89±6.02
6	19.45±0.12	170.89±3.61	139.91±1.05
7	15.81±0.15	152.83±2.10	121.70±0.71
8	13.01±0.25	132.91±1.06	106.54±0.64
9	14.03±0.15	150.57±0.71	123.75±0.15
Test No.	Isobutyryl shikonin	β,β-Dimethylacryl shikonin	2-Methylbutyryl
1	79.67±4.37	105.91±5.73	184.08±6.54
2	85.43±2.20	104.63±2.07	199.47±4.87
3	116.07±2.79	129.33±2.99	273.99±7.01
4	101.15±0.56	137.53±0.72	234.61±0.78
5	125.72±1.21	151.22±2.31	295.33±4.63
6	130.06±1.19	221.44±2.34	296.09±2.33
7	114.45±0.23	213.82±1.56	261.93±1.32
8	94.29±0.62	145.16±0.72	221.70±1.66
9	105.39±0.30	161.81±0.91	253.99±0.58

**图 3:**
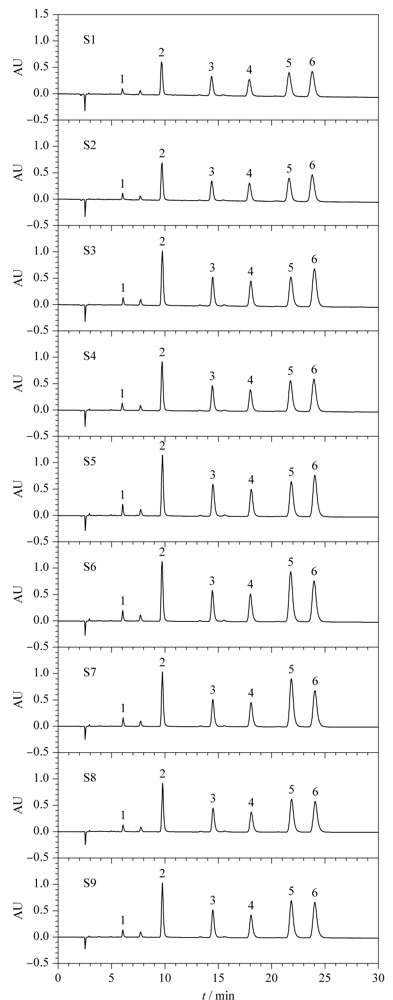
9组超临界流体萃取工艺制备所得紫草油的色谱图

影响超临界流体萃取有效成分的主要因素有萃取压力、萃取温度和CO_2_流量,本实验采用三因素三水平正交试验法来优化紫草超临界流体萃取的工艺条件,结果发现试验1至试验9所得的紫草油有效成分的总量分别是S1=562.59 μg/L, S2=604.64 μg/L, S3=818.74 μg/L, S4=738.42 μg/L, S5=909.84 μg/L, S6=977.84 μg/L, S7=880.54 μg/L, S8=713.61 μg/L, S9=809.54 μg/L。试验6的工艺条件所得的紫草油有效成分含量最高,且试验6所需的CO_2_流量最少,符合工业化大生产的需求。所以超临界流体萃取制备紫草油的最优工艺条件是:萃取压力23 MPa,萃取温度40 ℃, CO_2_流量27 L/h。

## 3 结论

本研究建立了HPLC-PAD法同时测定紫草油中6种有效成分含量的分析方法,并将此方法应用于超临界流体萃取制备紫草油的工艺优化过程,优化后的工艺条件能够满足紫草油制备需求,有效成分含量稳定。本方法简便可行、准确可靠,可用于超临界流体萃取制备紫草油的工艺过程和质量控制。
